# Viral E6 is overexpressed via high viral load in invasive cervical cancer with episomal HPV16

**DOI:** 10.1186/s12885-017-3124-9

**Published:** 2017-02-15

**Authors:** Die Hong, Jia Liu, Ying Hu, Xiaonan Lu, Baohua Li, Yang Li, Dongxiao Hu, Weiguo Lu, Xing Xie, Xiaodong Cheng

**Affiliations:** Department of Gynecologic Oncology, Women’s Hospital, School of Medicine, Women’s Reproductive Health Laboratory of Zhejiang Province, Zhejiang University, Xueshi Rd 1#, Hangzhou, 310006, China

**Keywords:** Cervical cancer, HPV16, Episome, Integration, Oncogene expression, Viral load

## Abstract

**Background:**

The integration of HR-HPV genome into host DNA is regarded as a key step for the development of cervical cancer. However, HR-HPV genome indeed exists as episome except for integrant. It may be alternative mechanisms in episome-associated carcinogenesis, although, by which HPV 16 episome induces cervical carcinogenesis is unclear now.

**Methods:**

Ninety-three invasive cervical cancer tissues with HPV16 positive were collected. Viral physical status was calculated from comparing E2 to E6-copies and detection of viral load was made with realtime-PCR using copy numbers of E6. HPV16 E6 mRNA transcript levels were measured by realtime-PCR. The methylation frequency of HPV16 promoter was detected by PCR and pyrosequencing.

**Results:**

In 93 samples, 21.5% (20/93) presented purely integrated viral genome, 53.8% (50/93) mixed viral genome, and 24.7% (23/93) purely episomal viral genome. Mean E6 expression in samples with purely episomal viral genomes was 7.13-fold higher than that with purely integrated viral genomes. Meanwhile, viral load in samples with purely episomal viral genomes was 4.53-fold higher than that with purely integrated viral genomes. E6 mRNA expression increased with the viral load in purely episomal cases. There were no differences of mean methylation frequency between purely episomal and integrated virus and among five CpG positions of HPV16 promoter for all samples. And there also was no correlation between E6 mRNA expression and methylation of HPV16 promoter among all samples with purely HPV16 episomal virus.

**Conclusions:**

HPV16 with the purely episomal viral genomes exists in a definite proportion of invasive cervical cancer, and episomal HPV16 also overexpresses E6 mRNA, probably through a high level of viral load.

## Background

Cervical cancer is one of the most common cancers among women worldwide. Infection with high-risk human papillomaviruses (HPV) is a causal factor for cervical intraepithelial neoplasia and cervical cancer. Among all the HR-HPV genotypes, HPV 16 is the most prevalent, reaching 65.2% of all genotypes in cervical cancer [1]. Human papillomavirus (HPV) genome integration into the host chromosome is considered as a crucial event during the life cycle of the virus and a major step towards carcinogenesis [2]. The integration of HPV16 DNA promotes a constitutive high expression level of E6 and E7 oncoproteins, resulting in the extensive proliferation of the infected epithelial cells. Typical integrants have complete or partial disruption of the open reading frame (ORF) in E2 ORF [3]. An important consequence of viral integration is an abolishment of E2 gene inhibiting the expression of oncogene E6 and E7, resulting in the extensive proliferation and malignant transformation of infected epithelial cells. Higher steady-state levels of viral oncogene transcripts in precancers and invasive cancers were expected as a consequence of virus integration. Thus, the integration of HR-HPV genome into host DNA is regarded as a key, even prerequisite, step for the development of cervical cancer.

However, the actual physical status of HPV 16 genome in cervical cancer cells appears far more complicated. For instance, Li et al. [4] reported the existence of HPV 16 integration in all of 15 cases of cervical cancer tissues, but Dutta S et al. [5] detected 82% cases contained HPV16 integrant in cervical cancer samples, and Mazumder D and his collogues found that 70.3% samples harbored integration HPV16 in cervical cancer tissue [6]. Further, Vinokurova and collaborators found that as high as 45% samples contained purely episomal virus in HPV16-positive cervical cancer tissues [7], and Cheung et al. [8] found pure episomal HPV16 genomes in 14 of 29 (48.3%) cervical cancer tissues. Therefore, HR-HPV genome indeed exists as episome except for integrant. This phenomenon points toward the biological plausibility of cervical carcinogenesis under the impact of HPV16 episome, in addition to E2 disruption due to viral genome integration into the host genome. It may be alternative mechanisms in episome-associated carcinogenesis, although, by which HPV 16 episome induces cervical carcinogenesis is unclear now.

Here, we detected HPV16 physical status, E6 mRNA transcript level, viral load, and E6 promoter methylation in HPV16 positive invasive cervical cancer tissues, and analyzed the association of HPV16 E6 expression with viral load and promoter methylation. The aim of the study was to understand the potential mechanism in cervical cancer pathogenesis with HPV16 episome infection.

## Methods

### Tissue sample collection

A total of 93 biopsied cervical cancer tissues were obtained from female patients (range 23–71 years, median 39 years) treated at the Department of Gynecology Oncology, Women’s Hospital, School of Medicine, Zhejiang University, China. All samples were histology-confirmed as invasive cervical cancer and had been confirmed to HPV16 infection by type specific PCR. Eighty-four cases of squamous cancer, three of adenosquamous cancer and six of adenocarcinoma were included. Two cases were stage IA1, 66 stage IB1, 13 stage IB2, ten stage IIA and two stage IIB. The study was done in accordance with the guidelines of the local ethical committee.

### DNA extraction, RNA extraction and reverse transcription

Biopsy tissues were physically disrupted by magnetic beads. DNA was extracted using UniversalGen DNA Kit (Cowin Biotech, Beijing, China) and stored at −80 °C until use. Total RNAs were extracted with TRIZOL reagent (Invitrogen Carlsbad, CA, USA) following manufacturer's instructions. cDNA was synthesized with PrimeScriptTM RT Master Mix (TaKaRa Otsu, Shiga, Japan) and stored at −80 °C until use.

### Detection of HPV16 physical status

To determine the physical status of the virus, E2 and E6 gene of HPV 16 DNA were quantified and the E2/E6 ratio was calculated. This approach is based on the fact that episomes present an identical amount of E2 and E6, whereas integration induces loss of E2.

Detection of E2 and E6 gene for HPV16 was performed with real-time PCR on the 7900HT Fast Real-Time PCR System. Standard curves for E2 and E6 gene were established by making a serial dilution of the plasmid pBR322 containing the total HPV16 genome. Dilutions were made to equal 50, 500, 5000, 50 000, 500 000 and 5000 000 copies of HPV16 E2 and E6. The sequence information of each primers used [9] (HPV16 E2F and E2R, HPV16 E6F and E6R) are available in Table 1. Samples were analyzed in triplicates and at least three no-template control reaction mixtures were included in each trial. After an initial denaturation at 95 °C for 15 s, reaction mixtures underwent 40 cycles at 95 °C for 5 s followed by 60 °C for 30 s. Results were analyzed by software 7900HT Fast System SDS Software. A ratio of ≥1 indicated pure episomal form, “0” indicated integration and a value bellow 1 indicated the presence of mixed infection of both integrated and episomal forms.

**Table 1:** The primer sequences for PCRs

Primers	Sequence
HPV16E6-F	5’-GAGAACTGCAATGTTTCAGGACC-3’
HPV16E6-R	5’-TGTATAGTTGTTTGCAGCTCTGTGC-3’
HPV16E2-F	5’-AACGAAGTATCCTCTCCTGAAATTATTAG-3’
HPV16E2-R	5’-CCAAGGCGACGGCTTTG-3’
HPV16MP-F	5’-TGTAAAATTGTATATGGGTGTGTG-3’
HPV16MP-Rbio	5’-(bio)ATCCTAAAACATTACAATTCTCTTTTAATA-3’
HPV16MP-S	5’-TTTATGTATAAAATTAAGGG-3’
GAPDH-DF	5’-TACTAGCGGTTTTACGGGCG-3’
GAPDH-DR	5’-TCGAACAGGAGGAGCAGAGAGCGA-3’
GAPDH-cDF	5’-GACAGTCAGCCGCATCTTCT-3’
GAPDH-cDR	5’-TTAAAAGCAGCCCTGGTGAC-3’

### Detection of HPV16 viral load

The strategy involved the measurement of the total viral load of HPV16 DNA by quantification of the HPV16 E6 gene and glyceraldehyde-3-phosphate dehydrogenase (GAPDH) with real-time PCR. The sequence information of GAPDH DNA primers (GAPDH-DF and GAPDH-DR) [10] were seen in Table 1. Standard curves for GPADH was obtained by amplification of a 2-fold dilution series of female human DNA between 112.5 and 1.75 ng/μL (Promega).

The amount of genomic DNA (ng) presented in each sample was divided by the weight of 1 genome equivalent (6.6 pg/cell) to obtain the number of cells in the sample [9]. Viral load was expressed as the copies of E6 per cell.

### Detection of E6 mRNA transcripts

E6 mRNA transcript expression was quantified by using the same primer. Human Endogenous Control GAPDH (GAPDH-cDF and GAPDH-cDR) was used as normalizer, listed in Table 1. Real-time PCR was detected under the same conditions with an initial denaturation at 95 °C for 15 s, reaction mixtures underwent 40 cycles at 95 °C for 5 s and followed by 60 °C for 30 s. Samples were also analyzed in triplicates in each trial.

### Detection the methylation frequency of HPV16 promoter

The Methylation frequency of HPV16 promoter was detected with PCR and pyrosequencing. The promoter P97 of HPV 16 contains potential methylation sites with five CpG dinucleotides located at 31, 37, 43, 52 and 58 nt (reference sequence NC_001526). Samples were treated with bisulphite using EZ DNA Methylation-Gold Kit according to manufacturer’s instructions (ZYMO Research, Irvine, USA). HPV16MP-F and HPV16MP-Rbio primers (Seen in Table 1) were used in PCR [11]. PCR conditions were as follows: preheating at 94 °C for 5 min, 40 cycles at 94 °C for 45 s, 55 °C for 45 s, 72 °C for 45 s, and a final extension at 72 °C for 10 min. One of the primers was labeled with 5-biotin. Then PCR amplifiers were detected by pyrosequencing with the primer HPV16MP-S. Samples were prepared for pyrosequencing using the Vacuum prep Workstation (Qiagen). Single-strand sequencing template was transferred to a 96-well sequencing plate containing sequencing primer HPV16MP-S (Seen in Table 1). The plate was incubated in 85 °C for 2 min. Pyrosequencing was performed in a PSQ 96 MA system using PyroMark Q96 Reagents (Qiagen). The results were analyzed in the Pyro Q-CpG Software to determine the proportion of C/T at the targeted position(s). Methylated samples were further divided into different methylation degree groups of highly methylated (>50%), medium methylated (11–50%) and low methylated (<10%).

### Statistical analysis

Data were analyzed with independent-samples *t*-test, Mann–Whitney test, Kruskal–Wallis test and Chi-Square test using IBM SPSS statistics 20. All P-values less than 0.05 were considered statistically significant. All reported P-values were bilateral.

## Results

### Physical status of HPV16 in invasive cervical cancer tissues

**Fig. 1:**
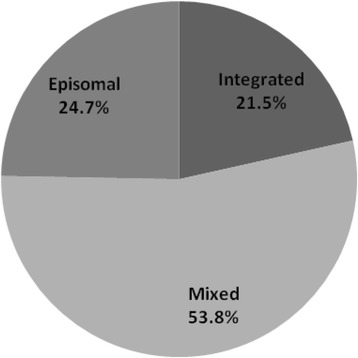
The physical status of three forms (episomal, integrated and mixed) in HPV16 genome was analyzed in 93 invasive cervical cancer samples. Of those, 21.5% presented complete integrated, 53.8% mixed, and 24.7% purely episomal viral genome, respectively

The physical status of all three forms (episomal, integrated and mixed) in HPV16 genome was analyzed in 93 invasive cervical cancer samples (Fig. 1). In detail, 21.5% (20/93) presented E2/E6 ratios of “0” and were regarded as complete integrated viral genome, 53.8% (50/93) presented ratios between “0-1” and regarded as mixed viral DNA, and 24.7% (23/93) presented ratios of “≥1” and regarded as purely episomal status.

### The expression of E6 mRNA and viral loads in different physical status from invasive cervical cancer tissues

We quantified E6 expression (normalized by GAPDH) by qRT-PCR of cDNA products generated directly from mRNA. Relative quantification based on comparative C_T_ method revealed the significant difference (*P* = 0.003; Kruskal-Wallis H test) among ∆C_T_ (E6 C_T_ - GPADH C_T_) values of cases with integrated viral genome (mean ∆C_T_ = 3.28), mixed viral genome (mean ∆C_T_ = 2.27) and episomal viral genome (mean ∆C_T_ = 0.45). The fold-change analysis (using 2^-∆∆CT^, where ∆∆C_T_ = mean ∆C_T_ of purely episomal - mean ∆C_T_ of purely integrated), depicted that E6 expression in cases with purely episomal viral genomes was 7.13 folds higher than that in cases with purely integrated viral genomes.

**Fig. 2:**
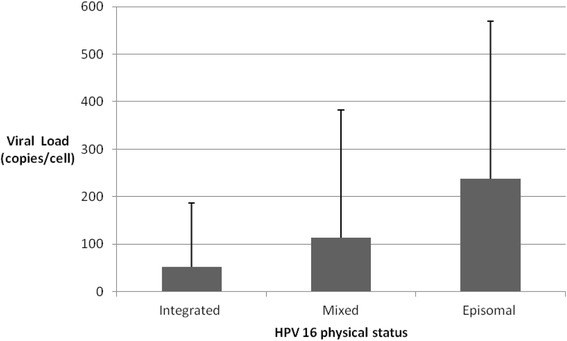
Mean viral load level of HPV 16 was 52.56 copies per cell with integration form, 113.65 copies per cell with mixed form, and 238.31 copies per cell with episomal form, respectively. Kruskal-Wallis H test showed that viral copy numbers per cell with episomal form were significantly higher than that with mixed form and purely integrated form (*P* = 0.002)

Viral load in the 93 samples varied between 0.85 and 1350.48 copies per cell with a median value of 18.60 copies per cell. Fig. 2 showed the distribution of viral load with respect to genome physical status. Mean viral load levels of HPV 16 were 52.56 copies per cell among integration form, 113.65 copies per cell among mixed form, and 238.31 copies per cell among episomal form. Viral copy numbers per cell were significantly higher (*P* =0.002; Kruskal-Wallis H test) among the episomal cases (mean 238.31 copies per cell) compared to the mixed cases (mean 113.65 copies per cell) and the purely integrated cases (mean 52.56 copies per cell). Viral load in cases with purely episomal viral genome was 4.53-fold more than that in cases with purely integrated viral genome.

Samples were further divided into two groups of high viral load group (≧50copies/cell) and low viral load group (<50copies/cell) on the basis of viral load. E6 mRNA expression was higher in high viral load group than that in low viral load group in purely episomal cases (*P* = 0.007; Mann–Whitney Test), but E6 mRNA expression was not significantly different between high and low viral load group in purely integrated cases.

### E6 mRNA expression and the methylation of HPV16 promoter from invasive cervical cancer tissues

**Fig. 3:**
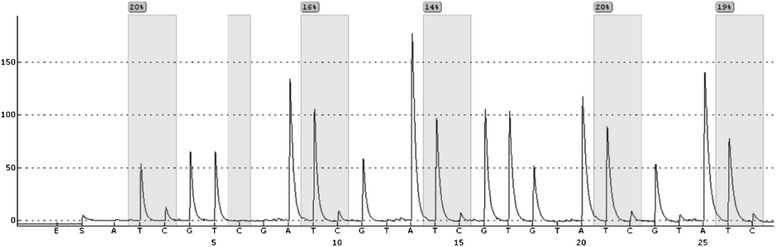
The promoter of HPV 16 E6 contains methylation sites with five CpGs dinucleotides located at 31 nt, 37 nt, 43 nt, 52 nt and 58 nt. After genomic DNA is treated with sodium bisulphate, T represents unmethylated C and C represents methylated C. The proportion of C represents the methylation frequency in the CpG dinucleotides. In this representative sequence, the methylation frequencies of the five CpGs at 31 nt, 37 nt, 43 nt, 52 nt and 58 nt were 20, 16, 14, 20, and 19%, respectively

Bisulfite modification and pyrosequencing were used to detect the five CpG methylation status of HPV 16 promoter (31 nt, 37 nt, 43 nt, 52 nt and 58 nt) in invasive cervical cancer tissues (Fig. 3). Out of 93 cases, examination of the promoter regions was unsuccessful in 3 cases, which were excluded from the statistical analysis. Therefore, 90 samples were analyzed for the methylation status of HPV 16 promoter, including 20 cases with purely integrated virus, 49 cases with mixed virus, and 21 cases with purely episomal virus.

**Fig. 4:**
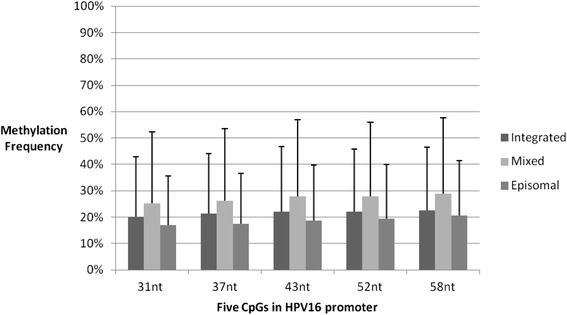
HPV 16 contains methylation sites with five CpG dinucleotides located at 31 nt, 37 nt, 43 nt, 52 nt and 58 nt. Methylation frequencies of the integrated, mixed and episomal virus were shown in the figure. There are no differences of mean methylation frequency among five CpG positions of HPV16 promoter

Mean methylation frequencies of the integrated, mixed and episomal virus were 20.2, 25.3, and 17.0%, respectively, at the 31 nt CpG; 21.5, 26.1, and 17.5%, respectively, at the 37 nt CpG; 22.1, 27.8, and 18.6%, respectively, at the 43 nt; 22.1, 28.0, and 19.3%, respectively, at the 52 nt CpG; 22.7, 28.8, and 20.5%, respectively, at the 58 nt CpG in HPV16 promoter. There were no differences of mean methylation frequency among the five CpG positions of HPV16 promoter for all cases (*P* > 0.05; Kruskal-Wallis H test), as shown in Fig. 4. Furthermore, there was no significant difference of promoter methylation frequency between purely episomal virus and purely integrated virus (*P* > 0.05; Kruskal-Wallis H test).

**Fig. 5:**
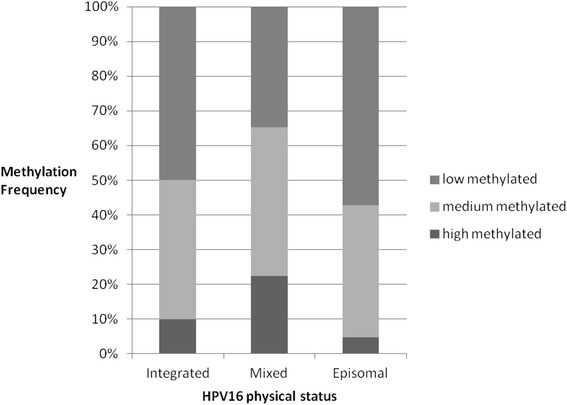
Methylation frequency is calculated from all five positions in the promoter as an average. >50% of methylation frequency is regarded as high methylated, 11–50% as medium methylated and <10% as low methylated. The proportions of highly, medium, and low methylation are shown in invasive cervical cancer tissues with integrated, mixed and episomal HPV16

Methylation frequency also was calculated from all five positions in the promoter as an average. In 90 invasive cervical cancer samples, 15.6% (14/90) were high methylated, 41.1% (37/90) were medium methylated, and 43.3% (39/90) were low methylated. No CpGs in the promoter had a methylation frequency of 100% in all invasive cervical cancer tissues. The proportions of highly, medium, and low methylated were 10, 40, and 50%, respectively, in the invasive cervical cancer tissues with integrated HPV16, while the proportions of these were 22.4, 42.9, and 34.7, respectively, with mixed HPV16, and 4.8, 38.1 and 57.1%, respectively, with episomal HPV16. The proportions of highly, medium, and low methylated HPV16 promoter were not significantly different between purely episomal virus and purely integrated virus, as shown in Fig. 5.

The correlation between the expression of E6 mRNA and methylation of HPV16 promoter was investigated. There were no differences in E6 mRNA expression among different methylation degrees of promoter in all 90 cases of invasive cervical cancer tissues. And there also was no correlation between E6 mRNA expression and methylation of HPV16 promoter among all samples with purely HPV16 episomal virus.

## Discussion

When high-risk human papillomavirus infects host cells, some of viral genomes integrated into host genome. As a consequence, deregulated expression of the HPV E6 and E7 genes in epithelial stem cells leads to malignant transformation in the respective cells at early stages of dysplasia. Integration of viral genome into the host genome, chiefly at fragile sites [2, 12], is considered as a critical event in the pathogenesis of cervical neoplasia due to the loss of negative feedback control of oncogene expression from viral itself through E2 disruption [13, 14]. In addition to viral oncogene over-expression, integrated HPV also may be regarded as a selectable form of the virus because this form not only is resistant to clearance from host, but also enables infected cells to maintain viral oncogene expression and avoids cell death [15].

However, HPV 16 also can exist as episome in clinical invasive cervical cancer tissues actually. Our results showed that a substantial proportion of invasive cervical cancer cases (24.7%) contained purely episomal viral genome, only 21.5% of invasive cervical cancer samples contained fully integrated genome, and 53.8% of samples had a mixed genome (integrated and episomal viral genome). Our study identified that a substantial proportion of individuals of invasive cervical cancer carried episomal virus, in line with previous studies [5, 8, 16]. Thus, our study suggest, together with other studies [8], that purely episomal viral genome can exist in invasive cervical cancer and integration is not a prerequisite step for invasive cervical cancer development.

It is known that high levels of the viral oncoproteins E6 and E7 in proliferating cells of epithelia are a necessity for oncogenic transformation. Up-regulation of E6/E7 expression also is constitutively required to maintain the transformed phenotype of invasive cervical cancer regardless of integration or episome status [8]. An in vitro experiment has revealed similarities between the episome and integrant associated routes of neoplastic progression [17]. Some reports have also showed that integration of the HPV16 genome does not invariably result in high levels of viral oncogene transcripts [18], and HR-HPV integration per se dose not necessarily lead to increased oncogene expression [17]. Our study found that purely episomal viral genome of HPV 16 in invasive cancer tissues also had E6 mRNA overexpression, which even higher than that of integrated virus, suggesting that HPV oncogenes are also overexpressed in invasive cervical cancer with viral episomal infection, but the mechanism may be involved in a way other than viral integration and E2 disruption.

It has been shown that viral load is one of the risk factors in invasive cervical cancer development [19]. Therefore, we detected viral load in different viral physical status. In our study, purely HPV 16 episome contained 238.31 copies per cell in invasive cervical cancer tissues, but purely integrated HPV 16 contained only 52.56 copies per cell. Mean viral load in cases with purely episomal viral genomes was 4.53-fold compared with purely integrated viral genomes. We further analyzed viral load and E6 mRNA transcript levels of HPV16 in different viral physical status, and found that mean E6 expression in cases harboring purely episomal viral genomes was 7.13-fold higher than those harboring purely integrated forms. Furthermore, our result showed that E6 mRNA expression increased simultaneously with the viral loads in HPV 16 episomal genomes, but not in HPV 16 integrated genomes. Similarly, Marongiu L and his collogues previously found that samples harboring solely episomal HPV16 DNA had a higher viral load than samples with solely integrated forms [20]. Thus, our and previously findings suggest that the overexpression of HPV oncogene may be mediated through a high viral load under HPV episomal status without E2 disruption [19]. In fact, intact E2 protein can enhance viral DNA replication by interacting with the viral replication factor E1 and recruiting it to the origin of replication [21, 22], and consequently facilitates viral genome segregation by tethering the viral genomes to host mitotic chromosomes [23].

Gene expression is influenced by promoter DNA methylation. HPV 16 E6 and E7 genes are transcribed from the promoter P97. The promoter P97 region contains several binding sites for cellular transcription factors, such as Sp1, TFIID, and E2BS. DNA methylation within the binding sites of transcription factors, such as Sp1, might block binding indirectly, either by changing the conformation of chromatin or by interacting with methyl-CpG-specific repressor proteins [24]. The capacity of the full-length E2 ORF gene product E2 to bind E2BSs in vitro is inhibited by methylation of cytosines within its binding site [25]. It is known that the interaction of E2 with various E2BSs mediates repression or activation of transcription and, through its interaction with E1, viral DNA replication [26]. However to date, few studies have examined the E6 mRNA transcript levels with respect to the promoter methylation of HPV16 in clinical invasive cervical tissues. Cheung JL and his collogues found that higher proportion of samples harboring the pure episomal form had high-level methylation at the 4 CpGs of two E2BSs in the promoter, compared to those of integrated forms (including mixed forms) [8]. However, our study did not find the differences in E6 mRNA expression between purely episomal and integrated virus and among different methylation degrees of promoter in the cases that containing purely HPV16 episomal virus. Thus, the impact of HPV promoter methylation on viral ocogene expression appears to be limited, but a further investigation with larger samples is needed.

## Conclusion

In summary, we found that HPV16 with the purely episomal viral genomes exists in a definite proportion of invasive cervical cancer, and episomal HPV16 also overexpresses E6 mRNA, probably through a high level of viral load. The impact of HPV16 E6/E7 promoter methylation on E6 expression may be limited in invasive cervical cancer with episomal HPV16 infection. Our results may provide another mechanism by which HR-HPV with episomal viral genomes induces the development of cervical cancer.

## Abbreviations

DNA: Deoxyribonucleic acid
GAPDH: Glyceraldehyde-3-phosphate dehydrogenase
HPV: Human papillomaviruses
HR-HPV: High-risk human papillomaviruses
mRNA: Messenger Ribonucleic Acid
ORF: Open reading frame
